# The Monitoring of Preoperative External Detorsion with Diffusion-Weighted Imaging in a Patient with Acute Testicular Torsion

**DOI:** 10.1155/2017/3702873

**Published:** 2017-08-28

**Authors:** Mehmet Beyazal, Fatma Beyazal Çeliker, Mehmet Fatih İnecikli, Maksude Esra Kadioğlu, Hasan Rıza Aydın, Tuğba Durakoğlugil

**Affiliations:** ^1^Department of Radiology, School of Medicine, Recep Tayyip Erdoğan University, Rize, Turkey; ^2^Department of Urology, School of Medicine, Recep Tayyip Erdoğan University, Rize, Turkey

## Abstract

Testicular torsion is one of the main causes of acute scrotum and may result in permanent damage of the testicular tissue. Color Doppler imaging has been frequently used in the diagnosis of testicular torsion and posttreatment follow-up period of the disease. There are some studies reporting the value and usefulness of diffusion-weighted imaging in the diagnosis of testicular torsion. However, to the best of our knowledge, there is no report regarding the monitoring of preoperative external detorsion in testicular torsion with diffusion-weighted imaging examination. In this article, diffusion-weighted imaging findings in the management of preoperative external detorsion in a case with testicular torsion were presented.

## 1. Introduction

Diffusion-weighted imaging (DWI) is a magnetic resonance imaging (MRI) technique which reflects the movements of water protons. Various pathologies such as tumor infiltration, inflammation, and ischemia affect the diffusion characteristics of the tissue via changing the histological architecture of the tissue, which results in signal differences on DWI. Many studies have reported that these signal changes provide useful contribution in the diagnosis and posttreatment follow-up for various organ and tissue pathologies [1, 2]. To the best of our knowledge, there are no available reports regarding DWI findings in the monitoring of preoperative external detorsion of acute testicular torsion. In this article, we presented the contribution of DWI in the diagnosis of testicular torsion and especially in the monitoring of preoperative external detorsion.

## 2. Case Report

**Figure 1:**
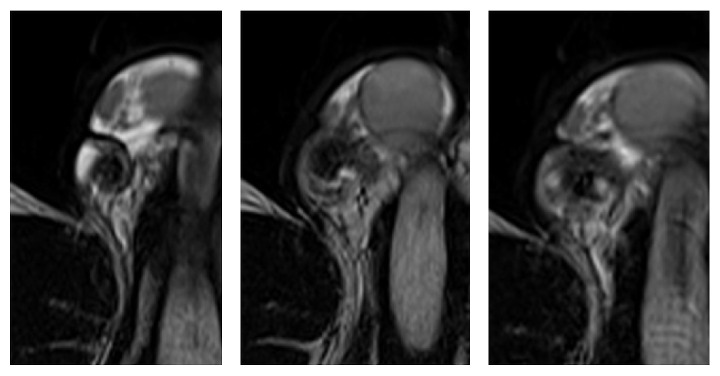
Axial T2-weighted images demonstrating right twisted spermatic cord.

**Figure 2:**
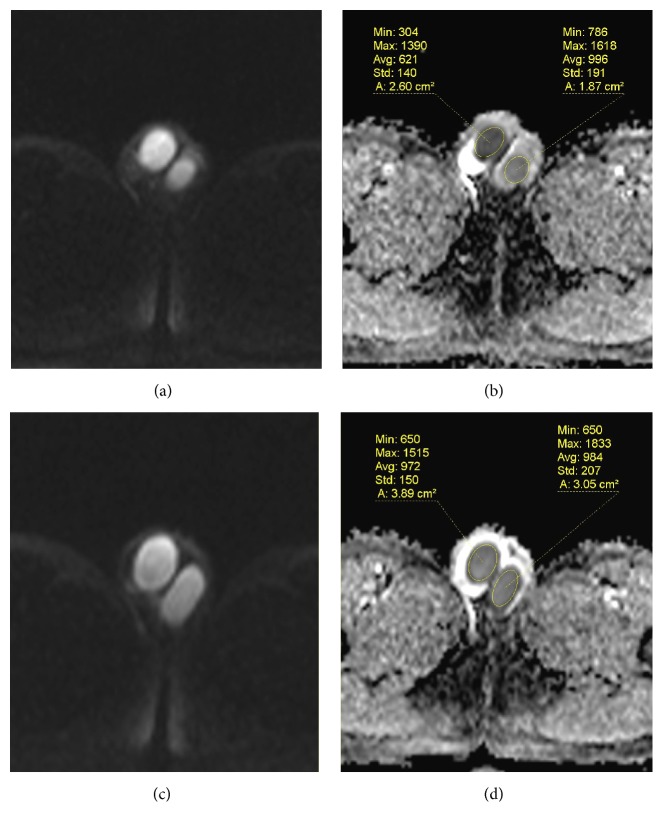
The right testicle shows high signal intensities on DWI (a). In the ADC map (b), the right testicle demonstrates lower ADC value than the left testicle. On DWI (c) and ADC map (d) obtained half an hour later following the preoperative external detorsion procedure, an isointense right testicle relative to the unaffected left testicle appears, which reflects the restoration of parenchymal blood flow.

A 15-year-old male patient was admitted to our clinic with a complaint of scrotal pain persisting for an hour. Physical examination revealed tenderness and painful right spermatic cord, epididymis, and testicle. There was no sign of scrotal erythema or swelling. The right testicle was located slightly above the oblique position. Laboratory examinations including liver, kidney, and urine analysis were all normal. On ultrasonographic evaluation, there was a heterogeneity and slight increase in the size of the right testicle. Although there was a normal parenchymal blood flow in the left testicle, color Doppler imaging revealed the absence of blood flow in the parenchyma of the right testicle. The patient immediately underwent axial T2-weighted and diffusion-weighted MRI. Magnetic resonance imaging examinations of the patient were performed with a 1.5 T MRI system. A multislice axial plane with a single shot, echo planar spin echo sequence (b: 50 sec/mm^2^, 400 sec/mm^2^, and 800 sec/mm^2^ and ADC) technique was performed for the DWI. Twisted right spermatic cord was observed on axial T2-weighted images (Figure 1). The right testicle had significantly increased signal intensity on DWI and low apparent diffusion coefficient (ADC) values compared to the left testicle (Figures 2(a) and 2(b)). The patient was diagnosed with testicular torsion based on clinical and radiological findings. Approximately 540 degrees of external manual detorsion was applied to the right testicle, which resulted in immediate relief of the patient's pain. After preoperative external detorsion, symmetrical parenchymal blood flow in the right testicle compared to the left testicle was observed on color Doppler imaging. MRI scan was repeated approximately half an hour later after the preoperative external detorsion and recovery of the twisted right spermatic cord was observed. The pathological signal changes in the right testicle on DWI and ADC maps detected before preoperative external detorsion disappeared and the signal intensities of the left and right testicles were almost equal (Figures 2(c) and 2(d)). The patient underwent transscrotal orchidopexy procedures the following day. The intraoperative findings demonstrated no twisted spermatic cord but revealed vascular congestion in the testicle and spermatic cord, which confirmed the diagnosis of prior torsion. The testicle was preserved and found to be viable. The testicle was attached to the scrotum using the appropriate method.

## 3. Discussion

Testicular torsion is one of the causes of acute scrotum, which may result in permanent damage of the testicular tissue. Color Doppler imaging has been frequently used in the diagnosis of testicular torsion and differential diagnosis of other acute scrotal disorders. It has been also reported that it could be used for the evaluation of testicular perfusion after the preoperative external detorsion in acute testicular torsion [3–5]. However, ultrasonography and color Doppler imaging are operator dependent and might yield inconclusive results [3].

DWI is a noninvasive imaging method reflecting the motion of water protons* in vivo*. The diffusion of water protons in tissue is affected by several factors including organization and cellular intensity of tissue and structure of extracellular distance [1, 2, 6]. Initially, DWI was performed to demonstrate cerebral infarctions. The apparent decrease in diffusion in cerebral infarction is attributed to fluid balance changes in intracellular and extracellular compartments due to massive ion and water influx [2]. There are many studies in the literature that have documented the use of DWI in the evaluation of various pathologies of organs and tissues. Also, Maki et al. reported that most cases of acute scrotum could be evaluated on the ADC map based on diffusion-weighted images and DWI of the scrotum can allow for the detection of testicular torsion [3].

Histologically, testicular ischemia has been shown to result in slight interstitial edema and blood congestion in the capillaries causing no changes in cellular morphology [3, 7]. In our case, these histopathological changes that occurred within the first few hours after torsion were determined as significantly different signal intensities in the affected testicle compared to the unaffected testicle on DWI. DWI images and ADC maps obtained an hour after the preoperative external detorsion revealed almost equalized signal intensities in the affected testicle compared to the unaffected testicle. These findings indicate that the testicular parenchyma was temporarily affected by acute ischemia.

In conclusion, this case report demonstrates that the monitoring of preoperative external detorsion of the spermatic cord with DWI could be a useful and valuable method in patients with acute testicular torsion. However, large sample studies are required to provide precise clarification and conclusions for this issue.
